# Community perception of barriers and facilitators to institutional delivery care-seeking behavior in Northwest Ethiopia: a qualitative study

**DOI:** 10.1186/s12978-022-01497-5

**Published:** 2022-09-20

**Authors:** Adane Nigusie, Telake Azale, Mezgebu Yitayal, Lemma Derseh

**Affiliations:** 1 Department of Health Promotion and Health Behavior, Institute of Public Health, College of Medicine and Health Sciences, University of Gondar, 196 Gondar, Ethiopia; 2 Departemenr of Health Systems and Policy, Institute of Public Health, College of Medicine and Health Sciences, University of Gondar, Gondar, Ethiopia; 3 Departement of Epidemiology and Biostatistics, Institute of Public Health, College of Medicine and Health Sciences, University of Gondar, Gondar, Ethiopia

**Keywords:** Facilitators to institutional delivery, Institutional delivery, Gondar, Ethiopia

## Abstract

**Background:**

Institutional delivery care-seeking behavior is considered a crucial step in preventing maternal and child death. However, the barriers and facilitators related to institutional delivery care-seeking behavior in the study area were not studied very in-depth. Hence, the current study aimed to explore barriers and enablers of institutional delivery care-seeking behavior in Northwest Ethiopia.

**Methods:**

A descriptive phenomenological qualitative inquiry was used. The data was collected by using a piloted interview guide. We collected data using in-depth interviews among women and men; and key informant interviews from the Female health development army and health extension workers in November and December 2019. Framework analysis was utilized for the analysis of the data.

**Results:**

This study found out the potential factors which hinder and facilitate institutional delivery. The barriers to institutional delivery included the belief that pregnancy and childbirth are normal and business of women’s, women’s preference of home delivery with traditional attendants, family influence, fear of bad behavior of health care workers, and lack of resources. The facilitators were free maternal services (ambulance services and maternity services), having the experience of safe childbirth at health facilities, and women’s health development army linkage with health extension workers.

**Conclusion:**

This study provides evidence that low-risk perception of home delivery, socio-cultural influences, service provision modalities, and financial factors were the determinants of institutional delivery service use. Therefore, a stage-matched intervention shall be designed to improve the uptake of institutional delivery service use.

**Supplementary Information:**

The online version contains supplementary material available at 10.1186/s12978-022-01497-5.

## Plain language summary

Institutional delivery plays a critical role in the prevention of maternal death and improves newborn health. Although most of maternal health services were free of charge in Ethiopia, institutional delivery is low in the rural Central Gondar zone of Wogera and East Dembiya districts. Low institutional delivery was the major contributors to high maternal deaths in Ethiopia. This low utilization of institutional delivery in the study area could also affect the health status of the newborn.

This study was conducted to explore the community perceptions of the socio-cultural and health service factors influencing institutional delivery. Overall, 30 interviews (18 In-depth and 12 Key-informants) were conducted in the selected rural kebeles of Wogera and East Dembiya districts to collect data from women and their husbands for in-depth interview; and key informant interviews from the female health development army and health extension workers.

The facilitators of institutional delivery categorized into three themes; namely, free maternal services (ambulance services and maternity services), having the experience of safe childbirth at health facilities, and women’s health development army linkage with health extension workers.

The socio-cultural factors impeding institutional delivery categorized into five themes; namely, the belief that pregnancy and childbirth are normal and business of women’s, women’s preference of home delivery with traditional attendants, family influence, fear of bad behavior of health care workers, and lack of resources.

Conducting a stage-matched intervention shall be designed to improve the uptake of institutional delivery service use in Wogera and East dembiya districts.

**Supplementary Information:**

The online version contains supplementary material available at 10.1186/s12978-022-01497-5.

## Background

Globally, a significant number of maternal deaths occur every year, even though the maternal mortality ratio (MMR) showed a substantial reduction in both developed and developing countries between 1990 and 2017. In 2017, 295,000 maternal deaths were recorded worldwide, and 86% of them were reported from low-income countries. Sub-Saharan Africa accounts for two-third (66%) of maternal deaths [1]. Even though a tremendous reduction in MMR (from 871/100,000 live birth to 471/100,000 live birth from 1990 to 2016) has been registered in Ethiopia, maternal mortality remains a challenge [2].

To end preventable maternal deaths in countries with high MMR (≥ 300 maternal deaths/100,000 live births), countries including Ethiopia, must emphasize the provision of comprehensive services of quality family planning, addressing direct causes of maternal deaths, improving care, and investing in basic infrastructures of the health care system [1, 3]. Giving birth at a health facility with the assistance of skilled health personnel is an effective and proven strategy to address direct causes of maternal death and avert maternal mortality. Introducing a robust referral system and accessing the necessary care could avoid a substantial proportion of maternal deaths [4–6].

In reducing maternal and neonatal mortality, giving birth at a health facility with the assistance of skilled health personnel is a very critical intervention. The accessibility of appropriate medical attention and a clean environment during delivery might reduce the risk of complications and infections [7], which might not be available at home. In Ethiopia, the percentage of institutional deliveries showed a significant increment from 26 to 48% between 2016 and 2019 [8]. However, this figure is lower as compared to the Health Sector Transformation Plan (HSTP) of Ethiopia in which the plan set to increase institutional deliveries attended by skilled health personnel to 90% [9].

The Ethiopian Federal Ministry of Health (FMOH) has been implemented different strategies, including; health extension program (HEP), a health development army (HDA), and Traditional birth attendants (TBA), to improve the institutional delivery-seeking behavior of women. The health Extension Workers (HEWs) have a role of assisting birthing women to move from home or health posts to health centers staffed with skilled birth attendants capable of managing complications [10]. The health development army (HDA) supports pregnant women to ensure the continuum of care from pregnancy to postnatal stage and supports the referral system by organizing ‘traditional ambulances’ to main roads or health posts [10]. The TBAs had the role of persuading women to start prenatal consultation as early as possible during pregnancy and to accompany women during childbirth to healthcare facilities [11].

Though the Ethiopian government has been exercising intensive efforts to resolve the problems with the utilization of delivery service and improve its uptake by the community, still utilization of delivery service was not satisfactory. To maintain effective primary health care coverage, the Ministry of Health has been investing significantly in the expansion and standardization of health facilities [12].

Nationally there is a struggle to increase institutional delivery service utilization. Since 2015, though access to primary health care services has improved, institutional delivery is still very low (31%) at the national level and the study area [12–14]. Even though there are several studies conducted in Ethiopia on institutional delivery service use and factors associated with it, there is a lack of evidence on the barriers and facilitators for institutional delivery service use in the context of the current study area (Central Gondar Zone, North West Ethiopia).

Thus, the present study was designed to explore barriers and enablers to improving the uptake of delivery service utilization among different community members (women with less than 1 year child, men, female health development army (HDA), and health extension workers (HEW)).

## Methods

### Study design

A transcendental/descriptive phenomenological study design was employed from November to December 2019.

### Theoretical framework

The descriptive phenomenology’s focus was to explore the correlation of the noema of experience (the ‘what’) and the noesis (the ‘how it is experienced’). Once ‘the things themselves have been identified, or otherwise analyzed, descriptive phenomenology considers its work done [15, 16]. The main purpose of phenomenological study design is to seek reality from individuals’ narratives of their perception, embody lived experience, feelings, and produce in-depth descriptions of a specific phenomenon, which starts and stops with lived experience. It should be a meaningful and significant experience of the phenomenon [17–19]. The goal of phenomenological study design is to describe the meaning of this experience both in terms of what was experienced and how it was experienced [20]. A qualitative inquiry is descriptive in nature; the nature of a phenomenon can be shaped and fixed by the investigators in question, which assists in exploring and describing the views held by people.

### Participant selection

The study group comprised key informants at the community level; and women who had at least one birth in the past year preceding the current study and their important others, i.e., their husbands at selected Kebeles (the lowest administrative unit in the country).

### Sampling

The intensity purposeful sampling method was used to recruit study participants for in-depth interviews (IDIs) and key-informant interviews (KIIs). The purpose of intensity purposeful sampling is to develop a comprehensive understanding of the barriers and facilitators to institutional delivery care seeking behavior. Therefore, participants of the IDIs were parts of the community with information-rich cases that manifest the phenomenon intensely [21], including; women who had at least one birth in the last year prior to this study and their important others. The sample of women was selected for in-depth interviews in consultation with the kebele administration and the HEWs using the maternal registration book, whereas participants of KIIs were HEWs and leaders of the female health development armies (HDAs). The purpose of key informant interviews is to collect information from a wide range of people, including community leaders (health development army) and professionals who live in the community (health extension workers) who have first-hand knowledge about the community. These community experts, with their particular knowledge and understanding, can provide insight on the nature of problems and give recommendations for solutions [22].

A list of potential key informants who are knowledgeable and closely linked to our population of interest was determined in consultation with the district health office. In creating this list, we were trying to get a diverse set of representatives with different backgrounds and from different groups or segments. This diversity of key informants provides a broad range of perspectives. If we only interview people of a particular background or segment, we may end up with one-sided or biased results. Interviewing key informants from a range of segments/parts/ of the community allows us to look at varying perspectives and underlying issues or problems.

### Method of approach

The study participants were approached by the principal investigator (AN) and one research assistant (RB) from the department of health education and behavioral sciences using a face-to-face interview in their homes. The principal investigator (AN) and research assistant (RB) had reach experience in qualitative research data collection and analysis.

### Sample size

The number of participants interviewed was determined by the information saturation criterion, which means that the information generated from repeated interviews becomes saturated [23, 24]. Thus, 18 participants for the in-depth and 12 participants for the key-informant interview were included in the study, and their informed consents were obtained.

### Non-participation

No individuals refused to participate in this study.

### Setting

The study was conducted in two rural districts of the central Gondar zone, which was purposefully selected among the 12 rural districts of the zone. Central Gondar zone is the newly established Zone of Amhara National Regional State (ANRS); its capital city, Gondar, is located 727 km away from Addis Ababa, the capital city of Ethiopia. The estimated population of the zone and the selected two rural districts was 2,288,442 million and 522,164, respectively, the majority of which are rural residents. The reproductive age group (15–49 years) women constitute 20.23% of the total population [unpublished Central Gondar zone report]; therefore, there are 105,635 women of reproductive age in the selected rural districts.

Based on the 2019 Central Gondar zone health department report, there were 14 districts (2 urban and 12 rural), 50 kebeles,75 health centers, and nine hospitals in the zone. According to the 2019 Mini-EDHS report, in the 5 years preceding the survey, institutional delivery coverage of the Amhara region was 54.2% [8].

### Setting of the data collection

The data were collected at the interviewee’s homes in a quiet, secure, and comfortable place with minimum sound disturbance and voice to maintain the quality of the recording and facilitate open discussion. The time and place of the interview were determined by interviewees.

### Presence of non-participants

Besides the participants and researchers, no one else participated in the study.

### Description of sample

The in-depth interviews participants were women who were residents of the study sites for a minimum of a year and had at least one birth in the year before this study; and their significant others. The key informants were female health development army leaders who were residents of the study area and had at least 12 months of HDA experience and those working as health extension workers in the study area for a minimum of 1 year.

### Data collection

#### Interview guide

We used a pilot-tested semi-structured interview guide prepared in English and translated to Amharic (local language of the study area) to elicit details of the data through probes. The interview guides for the IDIs and KIIs were developed separately based on literature related to the main research questions. The interview guides included eleven broad questions for the IDIs (Additional file 3) and eight for the KIIs (Additional file 4). Each guide was developed in the way that it captures the perception of the community on the barriers and enablers for institutional delivery.

#### Repeated interviews

Repeated interviews were not carried out.

#### Audio recording

The researchers used audio recording to collect the data.

#### Fieldnotes

Field notes were made by the investigators during and after the interview. We performed the field notes to document contextual information and contribute rich descriptive details about the context of statements made, supplementing the recorded and transcribed participants’ statements. In addition, we used field notes to clarify who the speaker was when recorded voices sound similar, and to describe changes in body language, long pauses, facial expressions, making or losing eye contact, or other events that can help interpret the meaning from the context of what was said.

#### Duration of the interview

The average duration of the interviews was one hour and 15 min time for each IDI and one hour and 20 min time for KIIs interviews.

#### Data saturation

The term data saturation, in the current study, refers to the point in data collection when new interviews produced little or no new information to address the research question. Based on the existing literature, a minimum of 12 interviews is typically needed [25].

#### Transcripts returned

The transcripts were not returned to participants for comments and corrections because the principal investigators had a prolonged engagement in the data collection process.

#### Data analysis

Two well-experianced researchers performed the data analysis. One of the researchers was a Ph.D. student in public health and an Assistance Professor of health promotion, and the second researcher was also an Assistance Professor of Health Education. Both researchers had taken training on qualitative data analysis methods using software and had experience in qualitative data analysis methods, teaching a course of qualitative research methods and analysis for Master program students, facilitating and delivering qualitative data analysis methods training for public health experts, lecturers and researchers.

Framework analysis was utilized for the analysis of the data. The framework method is most suitable for analyzing of interview data, where it is desirable to generate themes by making comparisons within and between them. This is an excellent tool for supporting thematic analysis because it provides a systematic model for managing and mapping the data.

The identification, isolation, and suspension of researchers’ preconceptions about the phenomenon under investigation (“bracketing”) were applied throughout the research process. Internal and external suppositions were also suspended. The researchers’ values, background, and cultural beliefs were maintained transparent, overt, and apparent. A reflexive diary was used to write down researchers’ thoughts, feelings, and perceptions to bring reflexivity into consciousness. It allows the researchers to re-examine their positions when issues were raised that might affect the research process.

#### Number of data coders

Two individuals have performed the coding independently after repeated readings of the transcribed documents.

#### Description of the coding tree

All tape-recorded data interviews and field notes were independently transcribed verbatim to Amharic (the local language) and then translated into the English language. The translated transcriptions were imported into ATLAS.ti8 software for the purpose of coding. Then the codebook (Additional file 1), with its definitions, was prepared in a separate word sheet document.

The analysis was performed by using the four theme development phases: 1. Familiarization with the data, 2. Re-visit research objectives, 3. Develop a framework, and 4. Identify patterns and Connections. Central themes were constructed based on the natural meaning of the categories. The investigators cross-cheeked the themes that emerged after analysis with the respective quotes. The findings were reported by a detailed description and interpretation of the meanings of the themes. Direct quotes of the participants were also included in the write-up of the findings to provide clear images for readers. The overall process of data analysis used an inductive approach, i.e., a data-driven coding process through the discussion of the researchers to identify themes. Finally, the study findings were reported based on the consolidated criteria for reporting qualitative research (COREQ) guidelines (Additional file 2).

#### Participant checking

The summary of the research findings was checked by the participants who provided relevant feedback.

#### Trustworthiness

The trustworthiness of this study was ensured through the following activities. Experts reviewed the interview guides to ensure the quality of the data, and a pilot test was done to ensure the flow of the questions and cultural sensitiveness. The investigators used simple language in conducting the interviews. An audit trail was considered a crucial technique to ensure the accuracy and credibility of the results. The researchers performed prolonged engagement with and persistent observations of research participants. The external audit served to confirm the accuracy of the findings and to ensure that the findings are supported by the collected data.

## Results

### Background information of the study participants

A total of 18 IDIs (nine men and nine women) and 12 KIIs (6HDA and 6HEW) were conducted among the selected kebeles of the Central Gondar Zone. Many of the key informants were within the age of 35–44 years and achieved secondary educational or above, whereas many participants of the in-depth interview participants were between 25 and 34 years of age and attained primary level education. All key informant participants were actively involved in the health care delivery system (Table 1).

**Table 1:** Socio-demographic characteristics of participants, Central Gondar zone, North West Ethiopia, 2020 (n = 30)

Characteristics		18 IDI		12KII	
		Female (n = 9)	Male (n = 9)	HDA (n = 6)	HEW (n = 6)
Age	25–34	4	4	2	3
	35–44	2	3	4	3
	≥ 45	3	2	0	0
Educational status	Illiterate	4	2	2	0
	Primary	2	5	1	0
	Secondary and above	3	2	3	6
Occupation	Farmer	9	9	6	0
	HEW	0	0	0	6

*IDI* in-depth interview, *KII* key informant interview, *HDA* Health Development Army

Many of the study participants reported that the utilization of delivery care services was low in their areas of residence. However, they mentioned that the utilization of health facilities for delivery had shown improvement compared to the previous years, and many women of the community want to have birth at a health facility especially, when they suffered pain after trying every possible home remedy.

**Fig. 1:**
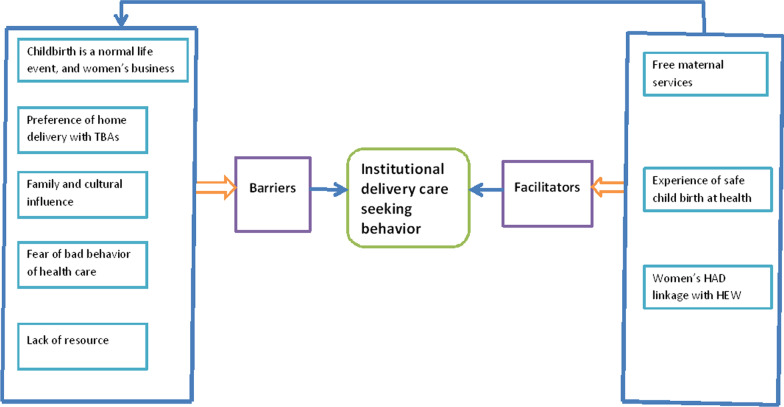
Visual framework analysis of the barriers and facilitators of institutional delivery care seeking behaviour, Central Gondar zone, North West Ethiopia

The participants also reported multiple barriers and facilitators for institutional delivery service utilization. The barriers were categorized into five sub-themes: (1) childbirth is a normal life event and business of women, (2) preference for home delivery with the help of a traditional birth attendant(TBA), (3) family and cultural influence, (4) fear of bad behavior of healthcare workers, and (5) lack of resources. The facilitators were categorized into three sub-themes: (1) free maternal services (ambulance services and maternity services), (2) the experience of safe childbirth at the health facility, and (3) linkage of women's HDA with HEW (Fig. 1).

### Barriers to institutional delivery care seeking behavior

#### Childbirth is a normal life event, and women’s business

As stated by the participants, the view of the community on childbirth had a fundamental role to play for institutional delivery service utilization. They explained that many relatives of women, including their husbands, believe birth is a normal life event with no risk and can be easily handled by relatives and TBAs at home. Trust in TBAs and previous experience of safe birth at home were considered as barriers. In addition, some women who had an experience of normal delivery at a health facility believed that delivery could be made without the support of a health professional at home.

A 29-year-old woman reported that

*“In my opinion, the main reason for home delivery (for not going to a health facility) is that most of the community, including me, thinks delivery is a normal event. Women listen to their mothers-in-law and mothers-in-law saying—we delivered a number of babies without a health facility at home safely while doing agricultural activity…”*

Another participant stated that,

*“There are mothers-in-law and husbands who don’t consider childbirth as a risk for mothers; rather, they believe it is a normal thing and can easily be managed at home at any time by the TBA and relatives without any cost in a healthy manner… so that the pregnant woman is supposed to do home and agricultural activities up to the date of delivery…” (*A 25-year-old woman’s HDA*)*

Pregnancy and childbirth are considered as the role of women by many of the community social norms. It was reported as the main reason for high levels of home delivery. As stated by the participants, they believe that there are no similar roles of women and men in giving care for labouring and pregnant women. Performing house tasks and providing body care for pregnant women were assumed to be the role of women by most of the participants.

*“I think that being pregnant and giving birth is a golden chance given for women by God, which is not for males. Therefore, it is a duty of women to give birth and we should keep our grandparents' tradition of giving birth....” (*A 55-year-old man*)*

*“If a pregnant woman goes to a health facility for the delivery service early, there may be a problem at home because she is the one who is going to manage home activities like feeding the family, keeping cows, preparing different home-based jobs,.especially those women who do not have t young female children, were suffering from such a critical problem… everybody, including the neighbor, assumes managing home activity and childbirth are roles for women….” (*A 38-year-old HDA*)*

### Preference for home delivery with TBAs’ care

The IDIs and KIIs focused on families and the community at large sharing opinions about institutional delivery service utilization. A common opinion was a worry about the culturally defined women’s activities at home, including deliveries at home without the help of skilled health personnel. Both female and male IDIs agreed with the reasons for home delivery over institutional delivery.

*“The TBA in our community has taken much training with several years of experience. She could take care of all the deliveries in our compounding, the most trustworthy one, and her hand is considered as a medicine for those mothers who are worried about the labour. She knows everything that should be done. She manages the delivery process without much pain by massaging the stomach using butter.” (*A 35-year-old woman*)*

*“Most of the time I wanted to go to a health center for delivery service. But I didn’t get the chance, because this child was delivered very easily due to a sudden onset of labour.” (*A 30-year-old mother who delivered at home*)*

The place of delivery might be decided based on the expected complications or the occurrence of an emergency in childbirth. As stated by the key informant, the community did not bring the labouring women to the health facility until the moment of labour pain. Even though women experienced an emergent complication, most women in the rural community did not go to a health facility for delivery at an early stage. This results in them being unable to reach the health facility for delivery care on time due to the short period of time because of the transportation problems, distance to health facilities, and short notice.

*“Since my house is so far from the main road, the vehicle or ambulance was unable to reach me in time, so I gave birth at home with the help of TBA Phone call was made to the health extension workers, HDAs, but [the] vehicle did not come, and [my] child was delivered at home.” (*A 35-year-old mother: home delivery*)*

*“The driver of the ambulance was not a good man. [The drivers] are so aggressive and arrogant that they switch their phones... At the hospital, they are sometimes available and sometimes not.” (A* 36-year-old HDA*)*

### Family and cultural influence

As stated by the participants, many of the relatives, including husbands, were involved more at the time of labour and childbirth care than at a time of regular follow-up as recommended by WHO for antenatal care (ANC) and postnatal care (PNC). This is because the process of regular follow-up of pregnancies is less stressful than childbirth, and every pregnant mother needs to have a regular ANC follow-up and skilled birth attendance. The study participants stated that most of the community members were guided by the ideas of relatives and neighbors for any services related to their pregnancy. Among the reasons, lack of awareness was mentioned as a reason for the community’s low level of readiness to institutional delivery care-seeking behavior. In addition, the lack of a warden at home for other family members, i.e., small children, expectant husbands, was also an impeding factor in accessing institutional delivery care.

*“No one takes over the home activity and so that they [pregnant mothers] can’t stay at the health center for five days before labour is coming in the health center waiting [to be able to go to their] home. Bringing birth on the road is common due to late traveling as a result of home activities, and then they [pregnant women] thought I would not go to the health center because it gives lots of trouble. They think of the small children at home.” (*A 30-year-old HDA*)*

*As I told you before...… “some of them [pregnant women] say they don’t want to give birth at the health cente.r, Even though, the women want to give birth at the health center, the men don’t want to do it., They[men] think we[HDA, HEW] will do other [necessary] things”…. (*A 33-year-old woman’s HDA*)*

The participant also stated that many members of the community didn’t worry about the risk of home delivery and were very happy with in-home delivery, especially those members of the community who live far from the main road.

*“There is a belief that nothing I will lose if I am delivering at home."There is no problem with home delivery. It is similar to the health facility….” (*A 35-year-old woman’s HDA*)*

*“Most of the communities want to hear the sound of a cock before going to a health facility; this is the commonest belief after animals get in their rooms, after the sun shines, after the sun rises, go to the health facility when the women lose their energy….” (*A 49-year-old head of female affairs*)*

*“When they [pregnant women] give birth, there is a problem with the cord tie. In my case, what happened when my wife got sick after she gave birth,we took her to the health center, and they referred us to Gondar for further examination, and they said there was a problem in the cord that is called in Amharic “Senge”, and after they diagnosed this, they took a kind of fluid from the back body, and after that, she became well, but those health professionals who worked here or at the health center didn’t treat this, and they didn’t diagnose it…. In addition, some people also go to traditional healers to avoid this. They use traditional herbals and they put it in the women’s bodies, and I think it treats the problem….” (*A 41-year-old kebele leader man*)*

Several participants also reported that some family members were happy with home delivery since they felt the freedom to perform different traditional activities for pregnant women if they had bled.

*“There is cultural influence; the older peoples make influences not to go to the health center. Some people go to witchcraft, everyone is not equal in terms of awareness, and there is men’s influence because… even when they see other pregnant women giving birth at health center well, they resist you [health development army]”…. (A 30-year-old, woman HDA)*

*“When the ‘serakiyan’ (bleeding) happens, in order to avoid this, they fire a bullet etc.….” (*A 41-year-old, woman*)*

### Fear of bad behavior of health care workers

Fear of the bad behavior of health workers was stated as a reason for the low utilization of health facility delivery with skilled health professionals by several participants.

Participants also said that the relationship between the health facility staff (health workers and non-health workers) and the community within the catchment area of the health facilities were not as good as expected. The participants said that some of the health workers had a negative attitude towards the community and did not serve them with respect. Few health workers were impolite and scared away mothers, making them deliver their babies at home.

This is explained by the following quotes:

*“There is one midwifery staff at our health center who is rude, arrogant, does not respect mothers, at times he beats mothers, refuses to give treatment to the patients and also speaks unethical things to the mothers about their clothing and personal hygiene in immoral ways….” (*A 27-year-old HEW*).*

*“There are few health workers (the male midwifery) are not cooperative and don’t want to touch our body and say why you don’t wash your body, you are offensive and have a bad smell…uffffffffff he says…. If you can, please dismiss him from this health center. Due to that, many women plan to give birth at home or in other health facilities by going far away if things are complicated unless our best option is at home” (*A 31-year-old woman*).*

### Lack of resource

The lack of resources in the community is one of the crucial barriers to institutional delivery service utilization. These include lack of income, food shortages at home and health facilities for family members who may go along with pregnant women, and user fees, including transportationcosts for family members.

Participants stated that the communities were not actively involved in the resource allocation for institutional delivery services; instead, they expect support from the government, including support for the food at the health facilities, transportation. This fact affected the women’s decision to deliver at the health facilities.

Although the government tries to coordinate the community to collect money and other necessary commodities for the women, including cereals, in order to arrange the availability of food at the health facilities, the community was not actively involved, and there was no sufficient food for all the family members who came to the facility with the pregnant mother. In addition, the government provided ambulances from women’s homes to the health facilities to alleviate the problem related to the transportation of pregnant women. Still, there is no transport service from the health center to women’s homes after giving birth, which hinders the community from choosing home delivery. In each health facility, women and their bystander attendants needed to make their food arrangements. It was difficult for women to have food during admission if they faced a lack of food at home or no one else at home prepared the food; therefore, when delivering in health facilities, such women could suffer from additional expenditures.

The quote below highlights the problem of food in health facilities and homes.

*“To have all deliveries at health facilities, you need to prepare the waiting home and distribute food not only for delivering mothers but also for their attendants. But currently, the attendants were not considered for the food as well as the waiting home so that women don't want to stay at a health facility.” (*A 40-year-old man*)*

*“We need an ambulance to give transportation services to return home after the delivery has been completed for both women who gave birth and the attendants. We cannot afford the current fee they charge us for using public transport. And they [drivers] have to be controlled to have a sense of server rather than showing bad behavior so that they [drivers] have to be punished for their bad behavior towards the pregnant women and other clients/attendants….” (*A 36-year-old HDA*)*

### Facilitators to institutional delivery care seeking behavior

#### Free maternal services

The participants reported that having free ambulance transportation had a substantial role in minimizing a birth on the road while the women traveled to the health facility. In addition, all services related to the pregnancy at the health facility were provided without any cost, including the maternal waiting home in few health facilities.

These interventions have improved the intention of women to utilize maternal and child health services, particularly for childbirth.

*“Currently, every maternal service is being given without any charge, health care for pregnant women is free, ….” (A* 27-year-old, HEW*)*

### Experience of safe childbirth at the health facility

Among many of the HEW tasks in the health extension program package, they are supposed to work in the community to increase health service utilization by improving the awareness and attitude of the large community. Moreover, they are the primary contact for the community and an essential bridge with health facilities. As stated by the participant, there were facilities that provided services in a suitable manner for the client; if women had experienced a well-coming approach by the health worker at the health facility and had received the services in a respectful manner, they could have had a positive feeling about the health facilities and their motivation to utilize delivery services would be high.

*“Having a birth in a health center had many advantages for the mother and the newborn. In the health center, the mother will be given an injection if she has bleeding but not at home (there is “Serakiyan” at home). The probability of death in the health facility is very low, which is safe because there is no much bleeding at a health facility; every necessary treatment facility will be given freely to her [mother]. When she [mother] is giving birth, the health workers clean the blood, moralized the family, and took her to another room, where all activities could be performed in a clean and attractive way without any frustration.” (*A 38-year-old woman*)*

Another KII participant stated that,

*“Bringing a birth at home or in a health facility is quite different from having a baby. When a woman gives birth in a health facility, there is no bleeding, no “serakiyan,” a very clean environment, a safe environment, no death, and no stress. A birth that is going to take place at home is very painful, unhygienic, not clean, high bleeding, no mechanism to stop bleeding; therefore, a high probability of death is there. Beds and cleaning materials such as soap and clean water are now available in health care facilities. Previously, nearly to 3 to 4 years ago, women were afraid to go to health facilities for the delivery service, but currently, this has somewhat improved, even though it needs further mobilization for the large community….”* (A 41-year-old HDA leader).

Previous interactions of people with healthcare settings impacted health behavior. This includes the experiences of women during the follow-up period and in the utilization of other health services, as well as family members’ experiences with the health care services.

*‘In the health facility, there is the safekeeping of health. Health professionals are available there. The availability of good-quality institutional delivery care and support from health care providers, the government, and free institutional delivery care services including transportation, medicine, and waiting for home services in the health facility...” (*A 29-year-old female HDA*).*

*“Previously, there were misperceptions like, for example, if we gave birth at the health center, we would acquire a communicable disease, etc.…But nowadays, going to the health center and giving birth there makes us happy because we will be given a soft drink like Mirinda to drink.” *(A 42-year-old woman).

### Women’s HDA linkage with HEW

According to the insights of the Ethiopian federal ministry of health, health extension workers are involved in different public health intervention programs, including improving the uptake of key maternal and child health services and creating demand for the large community by disseminating information and awareness about health services. Participants from IDIs and KIIs showed a favorable attitude towards women's HDA linkage of health extension workers and social mobilization, behavior change communication including educational activity they delivered at a different setting for the large population.

*“Most of the community has a favorable attitude towards women’s HDA and HEWs, in daily activity as well as the HEW and women’s HDA linkage. All the time they [HEW and HDA] come to our homes, they [HEW and HDA] mobilize, giving education without any salaries. They are eager to do work; they are visiting the pregnant woman at home and listing their name for the follow-up purpose. Any problem in the community could be managed at the home level by them [HEW and HDA]; they give advice to come to the health Post and, if necessary, to the nearest health center for follow-up….” (*A 47-year- old man*)*

If women had favorable attitudes towards the health facility and the health care providers within the health facility, that encouraged further attendance in the future,

*“Whenever the women are going to a health facility, if the health provider accepts the women with a good approach, i.e., respectful, greeting, and caring way, the probability of visiting the facility for the related services would be high and she could visit repeatedly….” (*A 37-year-old HDA leader*).*

Participants of KIIs also stated that the community needs to be sensitized periodically about the risk of the traditional birth attendant at home and the benefits of a skilled birth attendant for the newborn’s health, the mothers’ health, and the family at large in order to adapt to institutional delivery service as a habit in a sustainable way.

*“The community did not own the program [institutional delivery service utilization]… How do the children get home without their mother? We need leaders in this way. Cattle without a shepherd can’t be managed; “If the community is not mobilized, then there will be no work….” (*A 48-year-old kebele leader*).*

## Discussion

In this study, we explored the community perceptions of the barriers and facilitators related to institutional delivery of care-seeking behaviour. The study revealed considering pregnancy and childbirth as a normal life event and as a business of women; preference for TBA at home; family and cultural influences; fear of health care workers’ bad behavior; and lack of resources; as barriers to using institutional delivery services. Free maternal services, experience of safe childbirth at the health facility, and women’s HDA linkage with health extension workers were identified as facilitators to institutional delivery service utilization.

The findings of the study will help health professionals, health managers, administrators, and policymakers design strategies that could improve institutional delivery uptake. The findings of the study could be useful in different settings, in Ethiopia and beyond, with similar socio-political contexts [26].

The study showed that preference for home delivery was higher in remote areas and among marginalized communities. Such communities appear to trust and rely greatly on TBAs. Most rural communities in our study living in the most remote settings preferred TBAs, and in some cases, TBAs were the only option. This finding is contrary to the Ethiopian policy interventions that have shifted from the recognition of the TBA’s longstanding role in assisting women to birth to zero tolerance of women birthing at home with a TBA [27–29]. This is the problem with infrastructural shortages, i.e., for those communities who do not have access to roads and health facilities, the only option is TBAs and keeping all the cultural activities that their grandparents did for childbirth [30]. This finding implies that whenever the government or any other concerned body plans to address the full coverage of health services, the accessibility of the health facility needs to be considered for those communities who live in the hard-to-reach areas (villages far from the main road), i.e., the accessibility of the infrastructure should be addressed in all the villages, even within the same kebele.

The current study also revealed that there is a belief held by women and their relatives, who live in the study communities, that if complications occur during childbirth, women should get institutional delivery. However, the study further predicted that, if there are no complicated cases, community members consider institutional delivery is needless. Giving birth at home in front of their mothers, husbands, and mothers-in-law is considered as the best acceptable norm and has the most cultural influence on having a home delivery. This is consistent with the findings from Nepal, Indonesia, and Ethiopia [31–34]. This implied the need for educational programs to improve community belief and attitude regarding the benefits of health facility delivery services and the value of giving birth with the help of health care professionals for both the mother and the newborn.

Different traditional practices are undertaken before childbirth, at the time of childbirth, and after the birth of the child, including moving freely at the time of labour, no one touching the body other than her nearest relatives, having a coffee ceremony, enjoying traditional massages with the nearest relatives, and family influence, including overburdening of the woman by home activities like cooking food for the family, caring for small children at home, and caring for caws, were the most important reasons for women to have home delivery. This finding is in agreement with different other studies in different areas of low and middle-income countries like Nepal, Ethiopia, Laos [35–39]. This may be a cultural practice considered as a norm in most developing countries in the rural context [39]. This implies that any intervention mechanism designed for the prevention of home delivery should consider the norms and cultural practices held by the communities.

The current study also showed that women were not decision-makers regarding where to give birth; rather, they were supposed to follow the decision made by other family members. Most of the time, the decision-maker is mothers-in-law, husbands, and fathers-in-law, whose choice of place is home delivery, and the point of reference for the choice is their previous history of home delivery. This finding is contrary to a finding from Chitwan district, Nepal [40], in which institutional delivery was supported by most mothers-in-law. This discrepancy mainly arises because mothers-in-law in the current study area might not consider institutional delivery as essential, and pregnant women might be shy about communicating with their mothers-in-law [40].

The behavior of health workers, as well as privacy and confidentiality in health facilities, can have a significant impact on the preferred location of delivery. The current study shows that disrespect and abuse of women and their families like insulting, verbal abuse, bad facial expression, and discrimination based upon specific status or sex by the health care provider at a health facility is a deterrent to institutional delivery service utilization. This finding is in line with other studies [35, 41, 42]. This might be due to the low compassionate, respectful, and caring health care practices of health professionals, and a lack of community engagement of professionals, as well as a lack of professional autonomy and empowerment [43]. This finding predicts the need for more training and capacity-building programs for healthcare providers to help them have more compassionate, respectful, and caring health care practices. In addition, the community should be involved in the health facility's activities to have a sense of ownership and so that community to professional engagement can be improved and would have a strong linkage that helps the improvement of service utilization.

The lack of an organized waiting space or area in health facilities for labouring mothers' families, relatives, and neighbors who accompanied them to the health facility and some participants accounted for excluding friends from entry into the health facility compound. This could be a reason for women to ignore health facility delivery services and contribute to the low institutional delivery service uptake [44]. Besides, the lack of maternity waiting homes for all families in health facilities is reported to be a deterrent for women to use health services, particularly childbirth at a health facility with the help of skilled health personnel, which is supported by a finding from Zambia, Nepal, and Ghana [45–47]. The study participants stated that the lack of drop-back transportation services after childbirth at health facilities resulted in women's dissatisfaction and a gap in confidence and trust in the institutional delivery services.

The study's findings showed that free maternal services, including ambulances, were identified as facilitators for giving birth at a health facility. This study is in line with a study done in Uganda and Northern Ethiopia [48, 49]. The provision of free maternal related services substantially increased the use of institutional delivery service [48]. The government of Ethiopia is going to promote referral linkages between the community and health facilities through HEW referrals, provision of free ambulances, and improving basic emergency obstetric and newborn care in health facilities [50]. This implies that availing all maternal-related services is a critical strategy to avert home delivery in a sustainable way.

The current study found out that female HDA and HEWs conducted active surveillance of early pregnancy identification to improve uptake of ANC and institutional delivery service. This assisted the health care providers to compute pregnant women's estimated date of delivery, provide essential obstetric health care appropriate to their gestational age, and be able to conduct close follow-up and care during pregnancy, delivery, and postpartum. Conducting active surveillance of early pregnancy identification through female HDA and HEWs was found to have improved uptake of institutional delivery service [49].

Female HDAs had a significant impact on improving service utilization behavior in their community. They delivered important health messages and information to pregnant women, identified pregnancies and linked them with HEWs, mobilized pregnant women for the conference, notified HEWs of labor, and arranged ambulance service [49]. The engagement of female HDA in maternal and newborn health increased the need for maternal and child health service utilization, and to have better birth outcomes [51, 52].

The current study found that female HDA linkage with HEW has a significant role in conducting pregnant women’s conferences, which helps to improve awareness of pregnancy danger signs and birth preparedness, promote health-seeking behavior, and increase uptake of delivery service utilization. This result was in agreement with research done in Ethiopia [52]. The awareness creation program conducted by female HDA and HEW via pregnant women's conference improved mothers' awareness of pregnancy danger signs, birth preparedness and complication readiness, and utilization of institutional delivery service [53]. This implies that having a frequent and strong awareness creation program for pregnant women and their relatives is key for the improvement of health facility delivery service utilization. The readers and consumers of this research will be able to make inferences about extrapolating the findings to other new settings. In addition, it helps in designing an intervention at the local level to improve uptake of delivery services and contributes towards improving the readiness level of the local community for the promotion of delivery services.

### Strengths and limitations

An inclusive view of community perception of the barriers and enablers of institutional delivery of care-seeking behavior has been obtained from different categories of the community. This reflects a wide range of experience to design appropriate interventions for the barriers. As a limitation, the findings of this study can be applied to the specified study area only; they can not be taken to apply to another area.

## Conclusions

The current study has identified childbirth as a normal life event and business orf women; preferring home delivery with TBA; family and cultural influence; fear of bad behavior of health care workers; and lack of resources as barriers, whereas free maternal services, experience of safe childbirth at the health facility, and women’s HDA linkage with HEW were the enablers of institutional delivery service utilization.

From this study we suggest: (1) strengthening community engagement and establishing awareness creation programs via health promotion programs to have equal sharing of responsibilities for home management and child care between males and females; (2) infrastructure provision could improve utilization of institutional delivery care services by ignoring TBAs, especially for communities living in remote villages; (3) To ensure the sustainability and accessibility of institutional delivery care seeking behavior, there should be an inclusive approach from all the concerned bodies and also strengthening the women’s HDA linkage with HEWs; (4) Health care workers must provide health care in a compassionate, respectful, and caring manner, as well as develop good interpersonal communication skills in a culturally sensitive manner; (5) Any intervention program should consider empowering women in decision-making about where to deliver and resource management; and (6) Since TBAs are still the only option for assisting in the delivery process for communities living far from the main road, it is better to thank them for their valuable contribution rather than insult and criticize them. Because they were highly respected by the large community, they could easily change the attitude of the community to have institutional delivery service utilization if they considered the remote villages.

## Supplementary Information

**Additional file 1.** Code book.

**Additional file 2.** COREQ guideline.

**Additional file 3.** IDIs interview guide.

**Additional file 4.** KIIs interview guide.

## Data Availability

The datasets generated and/or analyzed during the current study are available from the corresponding author (Adane Nigusie- e-mail adane_n@yahoo.com) on reasonable request as this is part of a Ph.D. work.

## Abbreviations

ANC: Antenatal care
EDHS: Ethiopian Demographic Health Survey
HDA: Health development army
HEW: Health extension workers
HW: Health workers
IDI: In-depth interview
KII: Key informant interview
HW: Health workers
PNC: Post natal care
TBAs: Traditional birth attendances
WHO: World Health Organization
