# Supplementary material for: Community perception of barriers and facilitators to institutional delivery care-seeking behavior in Northwest Ethiopia: a qualitative study
Source: Reprod Health. 2022 Sep 20;19:193. doi: 10.1186/s12978-022-01497-5 (PMC9487075; doi:10.1186/s12978-022-01497-5)
Supplement: Supplementary file 1 — Additional file 1. Code book. [file 12978_2022_1497_MOESM1_ESM.pdf]

Title of the manuscript:

Community perception of barriers and facilitators to institutional delivery care-seeking behavior in North West Ethiopia: a qualitative study

Code-book *(Supplementary document 1)*

Theme	Sub-theme	Code	Code definitions
**Barrier to institutional delivery**	Pregnancy and child birth is normal life event and women's business	Pregnancy is not risk	The respondents reported that the community/they/ is not fear about the risk of pregnancy
		Pregnancy and child Birth is the concern of women	The respondents reported that the community/they/ considers pregnancy and child birth is for women ,male has not any concern
		Pregnancy is normal life	The respondents reported that the community/they/ is thinking pregnancy as a normal life
		Child birth is normal life event	The respondents reported that the community/they/ is child birth as a normal life
	Preference of home delivery with TBAs care	Home delivery	The respondents reported that the community/they/ prefers delivery at home rather than health institutions ire-regardless of the reason
		Assisted by TBA	The respondents reported that the community/they/ birth at home assisted by TBA
	Family and cultural influence	Place of delivery decide by women	The respondents reported that mostly the place of delivery in the community decided by the women
		Place of delivery decide by family members	The respondents reported that mostly the place of delivery in the community decided by any family members other than the women
		Cultural practice at home for labor	The respondents reported that the community including the women gives emphasis for the cultural practices at home during labor.
		Home based activity(home management)	The respondents reported that the community including the women highly burdened by home care activity like feeding the children, oxen, cows, hens….
	Fear of bad behavior of healthcare workers	No respecting by the health workers	The respondents reported that the health care workers at health facility wherever is it showing disrespectful activity for women or their family
		Insulting	The respondents reported that the healthcare workers at health facility insulting the women or any relatives at health facility
		Being ignorant for greetings	No good well coming approach at health facility including the administration staffs at health facility
		Less caring behavior	No appropriate caring for the women as well as their families
		Bad facial expression	The facial expression of the health care workers and administrative staffs seems to angry
	Lack of resource	Shortage of transport fee	No enough money for transportation of the women and their families
		Lack of waiting home	No enough waiting home at health facility for the

			women including their family
		Lack of food at health facility	No enough food at health facility for the women as well as their families
		Lack of money	No e money at the hands of women, searching from husbands…
**Facilitators to institutional delivery**	Free maternal services	Presence of Ambulance	There is ambulance services for pregnant women transportation
		Presence of waiting home	There is enough waiting home at health facility to stay until labor
		Free maternity services	All services relating to the pregnancy is free of fee (drug, examination, diagnosis…).
	Perceived experience of safe child birth at health facility	No-bleeding at health facility	Birth at health facility is safe and no worried about the bleeding; it can be easily managed by the health worker.
		Clean and hygienic service at health facility	Birth at health facility is hygienic and clean, no contamination, every equipment is safe and free from sharing.
		Less pain labour at health facility	Labour is much less at health facility, no massage as local…
		Birth at health facility is safe for the baby and women	The women and the baby would be safe if the birth is at health facility with the help of health workers.
		All services at health facility is free	Every services at health facility is free from any cost, no fee for pregnancy and child birth related services
	Women's HDA linkage with HEW	Follow-up of pregnant women	Following the status of pregnant women with in the specified working village
		Women HDA discussion	Conducting periodical discussion about the status of the pregnant women with HEW and them selves
		Surveillance of pregnant women	Active surveillance of new pregnant women with in the catchment villages
		Commitment of HDA	Serving the community by taking a sense of ownership
		Broad base	Able to standing alone without any incentives to do the capacity building and mobilization of the large community
		Communication	Making a smart interpersonal communication with the large community
