# Supplementary material for: Community perception of barriers and facilitators to institutional delivery care-seeking behavior in Northwest Ethiopia: a qualitative study
Source: Reprod Health. 2022 Sep 20;19:193. doi: 10.1186/s12978-022-01497-5 (PMC9487075; doi:10.1186/s12978-022-01497-5)
Supplement: Supplementary file 2 — Additional file 2. COREQ guideline. [file 12978_2022_1497_MOESM2_ESM.pdf]

Title of the manuscript:

Community perception of barriers and facilitators to institutional delivery care-seeking behaviour in Northwest Ethiopia: a qualitative study

The consolidated criteria for reporting qualitative research (COREQ) guidelines (***Supplementary document 2***)

No. Item	Guide questions/description	Reported on Section
**Domain 1: Research team and reflexivity**		
Personal Characteristics		
1. Interviewer/facilitator	Which author(s) conducted the Interview or focus group?	Adane Nigusie<br>Telake Azale<br>Mezgebu Yitayal<br>Lemma Derseh
2. Credentials	What were the researchers' credentials? E.g. PhD, MD	AN: MPH, PhD candidate<br>TA: PhD<br>MY: PhD<br>LD: PhD
3. Occupation	What was their occupation at the time of the study?	AN and TA are researchers in the field of health behaviour and health promotion.<br>MY is researcher in the field of Health Systems and Policy<br>LD is researcher in the field of Epidemiology and Biostatics
4. Gender	Was the researcher male or female?	All researchers are male.
5. Experience and training	What experience or training did the researchers have?	The researchers have training in qualitative and quantitative research methods in health promotion and public health. All are experienced in research and conducted many researches and published in international reputable journals.
Relationship with participants		
6. Relationship established	Was a relationship established prior to study commencement?	No author knew had relationship before the interview it was for the first time during these interviews.
7. Participant knowledge of the interviewer	What did the participants know about the researcher? e.g. personal goals, reasons for doing the research	Participant were informed about the research aims on consent form and information sheet and beside that before starting interview the reason of the research were explained.
8. Interviewer characteristics	What characteristics were reported about the interviewer/facilitator? e.g. Bias, assumptions, reasons and interests in the research topic	Methods (*Method of approach page 8*)
**Domain 2: study design**		
Theoretical framework		
9. Methodological orientation and Theory	What methodological orientation was stated to underpin the study? e.g. grounded theory, discourse analysis,	Methods (*page 6)*

	ethnography, phenomenology, content analysis	
Participant selection		
10. Sampling	How were participants selected? e.g. purposive, convenience, consecutive, snowball	Methods(Sampling page 7*)*
11. Method of approach	How were participants approached? e.g. face-to-face, telephone, mail, email	Methods *(Method of approach page 8)*
12. Sample size	How many participants were in the study?	Methods *(Sample size page 8)*
13. Non-participation	How many people refused to participate or dropped out? Reasons?	No individuals refused (page 8).
Setting		
14. Setting of data collection	Where was the data collected? e.g. home, clinic, workplace	Methods *(Setting of data collection page 9)*
15. Presence of non-participants	Was anyone else present besides the participants and researchers?	No (page 9)
16. Description of sample	What are the important characteristics of the sample? e.g. demographic data, date	Methods (Description of sample page 9)
Data collection		
17. Interview guide	Were questions, prompts, guides provided by the authors? Was it pilot tested?	Methods *(Interview guide page 10)*
18. Repeat interviews	Were repeat inter views carried out? If yes, how many?	No (page 10)
19. Audio/visual recording	Did the research use audio or visual recording to collect the data?	Methods *(Audio recording page 10)*
20. Field notes	Were field notes made during and/or after the interview or focus group?	Methods *(field notes page 10)*
21. Duration	What was the duration of the inter views or focus group?	Methods *(duration page 10)*
22. Data saturation	Was data saturation discussed?	Methods *(data saturation page 11)*
23. Transcripts returned	Were transcripts returned to participants for comment and/or correction?	No (Page 11)
**Domain 3: analysis and findings**		
Data analysis		
24. Number of data coders	How many data coders coded the data?	Methods *(page 11)*
25. Description of the coding tree	Did authors provide a description of the coding tree?	Methods(page 12)
26. Derivation of themes	Were themes identified in advance or derived from the data?	Methods (page 12)
27. Software	What software, if applicable, was used to manage the data?	Methods *(page 12)*
28. Participant checking	Did participants provide feedback on the findings?	Methods (page 13)
Reporting		
29. Quotations presented	Were participant quotations presented to illustrate the themes/findings?	Results
30. Data and findings consistent	Was there consistency between the data presented and the findings?	Discussion
31. Clarity of major themes	Were major themes clearly presented in the findings?	Results
32. Clarity of minor themes	Is there a description of diverse cases or discussion of minor themes?	Results
