# Supplementary material for: Community perception of barriers and facilitators to institutional delivery care-seeking behavior in Northwest Ethiopia: a qualitative study
Source: Reprod Health. 2022 Sep 20;19:193. doi: 10.1186/s12978-022-01497-5 (PMC9487075; doi:10.1186/s12978-022-01497-5)
Supplement: Supplementary file 4 — Additional file 4. KIIs interview guide. [file 12978_2022_1497_MOESM4_ESM.pdf]

Key informant In-depth interview Questionnaire guide

Community perception of barriers and facilitators to institutional delivery care-seeking behaviour. An information sheet used to ask permission.

Key informant In-depth interview guide question with female health development army leaders (HDAs) and health extension workers (HEWs).

Information sheet: Read the statements to the respondent

Purpose of the research project: Dear Participants, The University of Gondar Institute of Public Health and Principal Investigator (Adane Nigusie) are conducting the study in rural districts of Central Gondar zone among selected District and kebele, Northwest Ethiopia. This study is aimed **to explore barriers and enablers to improving the uptake of delivery service utilization.** You have been chosen to participate in this study.

Procedure: In order to collect our data, we invite you to take part in our project. If you are willing, you need to understand and sign the consent form. We will ask some questions.

Risk and /or discomfort: By participating in this research project you may feel some discomfort especially on sacrificing your time otherwise no risk in participating in this study.

Benefits: If you are participating in this research project, the output of the study will have both direct and indirect benefit to you, as well as your family and the community at large will get services in the future.

Incentives/payments for participating: You will not be provided any incentives or payment to take part in this project.

Confidentiality: The information collected from this research project will kept confidential and information about you that will be collected by this study will be stored in a file, without your name, but a code number assigned to it. And it will not be revealed to anyone except the principal investigator and will be kept locked with key.

Right to refusal or withdraw: You have the full right to refuse from participating in this research. Your refusal will not affect you from getting any kind of health related service.

Person to contact: If you want to know more information you can contact; Mr. Adane Nigusie by Tel: mobile +251-913673758 IRB of UOG by Tel: 251-581141231 If you agree to participate in the study described above, please sign below and write the date (in the presence of other family members). Please answer every question in the booklet. Instructions for how to respond to the different questions in the booklet are provided at the top of each page.

Consent Form

I, the undersigned, have understood the objective of the project "**to explore barriers and enablers to improving the uptake of delivery service utilization in rural districts of Central Gondar zone among selected District and kebele, Northwest Ethiopia**.", and agreed to be included in the study as explained by the researchers.

For this, I agree to participate in these in-depth interviews. I also agree to the use of anonymous quotes and understand that these quotes will not be directly traceable to me. With due understanding of the aforementioned information, are you willing to participate in the study?

INFORMED CONSENT FORMS
I_____________, the member of the research team would like to inform you that the purpose of this interview is to explore barriers and enablers to improving the uptake of delivery service utilization. Your accurate responses are vital for the quality of our research outputs. Your responses are confidential. By participating in this research there is no potential harm/benefit.<br>Are you willing to participate: Yes_______ No______<br>If you are willing to participate Please put your Signature: _____________.<br>No (Terminate the interview) Interviewer Name __________Signature ________date ______________

Key informant In-depth interview guide question with women female health development army leaders and health extenstion workers

**Basic socio-demographic characteristics**	
Interviewer : ________________________	Interviewee code: ___________________<br>Age: __________________Sex:________<br>Occupation: _____________________<br>Educational level:________<br>Attention given for this interview:<br>☐ 1. HDAs leader<br>☐ 2. HEWs
**Date: dd/mm/yy:** _ _ _ _ / _ _ /_ _	Have you agree for the interview:<br><br>☐ 1. Yes ☐ 2. No
Started time of the interview: ____:____	Completed time of interview: ____:____
Recorder ID:________________________	Recording #______________________

**Introduction**
❖ We are gathering today because we are interested in learning more about institutional delivery utilization of the community from you. We are specifically interested in learning about concerns that related to the barriers and enablers of institutional delivery care seeking behaviour and perception of the community. We are going to talk about each of these topics. To make you more comfortable, you do not need to share what you experience specifically. You can react what you know may be concerns for other women / men like you.
**Opening: Ice-Breaker**
To get started, we would like to have you to share something about yourself. You do not need to say your name. Please go around and share the part of the day you enjoy the most or anything you like.

Detail question guide for key informant in-depth interview (HDAs and HEWs)	
Questions	Probing questions
1. What is the status of institutional delivery currently?	➢ The coverage and utilization of Institutional delivery, Trends in institutional delivery care seeking behaviors.
2. What are the common practices the community have before and during delivery; and underlying reasons	➢ What is the preference of place of birth in the community?
3. How do you explain the feeling of the community for institutional delivery	➢ Does the community strengthening institutional delivery, role of family members, Roles of community members, i.e. TBAs, HEW, neighbors?
4. What do you think is the barrier for using institutional delivery? (What are/were some of the barriers if any, that you see?)	➢ Lack of key support? Lack of technical assistance? Staff turnover? Community perception? Culture presence of TBA? Fear of Money? Distance of HF? Strengthening…)<br>➢ How did you overcome the barrier(s)?
5. What do you think is the facilitators for using institutional delivery?	➢ Enabling factors, how do you look the facilitators?
6. What do you recommend for the continuity of the service i.e. institutional delivery care seeking behaviour at health facility?	➢ What things you recommend to be removed from home delivery free packages strategy?<br>➢ Please provide a justification for your response.

7. What factors do you think affect the health care seeking behaviour of the community?	➢ Do concerns about quality of care/treatment by health posts prevent use of services? Do costs or travel distances limit use of services? Do lack of knowledge about when to see care barrier to use of services?
8. In your view, in the future, what would be the most effective way to improve the institutional delivery care seeking behaviour of the whole community at large?	➢ Probe for suggestions for addressing community themselves, as well as other important household decision-makers.

Thank you for answering all our questions!!!

Maybe you have thought of something that we have left out. Is there anything else that you'd like to tell me/ us about your experience regarding institutional delivery?
